# Non-stimulant pharmacotherapy for ADHD in adults with comorbid substance use disorders: a review of randomized trials

**DOI:** 10.1192/j.eurpsy.2026.10493

**Published:** 2026-07-31

**Authors:** S. Mizera, K. Krysta

**Affiliations:** 1 Doctoral School, Department and Clinic of Psychiatric Rehabilitation, Faculty of Medical Science in Katowice, Medical University of Silesia; 2 Department and Clinic of Psychiatric Rehabilitation, Faculty of Medical Science in Katowice, Medical University of Silesia, Katowice, Poland

## Abstract

**Introduction:**

Attention-deficit/hyperactivity disorder (ADHD) frequently co-occurs with substance use disorders (SUD) in adults, complicating care and raising concern about stimulant misuse. Non-stimulant medications are considered safer, yet their efficacy and impact on SUD outcomes remain uncertain.

**Objectives:**

To synthesize randomized controlled evidence on the efficacy and safety of non-stimulant medications for adults with ADHD and comorbid SUD.

**Methods:**

We conducted a review of randomized controlled trials evaluating non-stimulant pharmacotherapies (e.g., atomoxetine, bupropion, α2-agonists, viloxazine) in adults with ADHD and SUD, including alcohol (AUD), cannabis (CUD), opioid (OUD), and stimulant (StUD) disorders. Primary outcomes were ADHD symptom change and SUD-related outcomes (abstinence, relapse, substance use/biomarkers, craving, treatment retention). Safety, misuse, and diversion were assessed qualitatively.

**Results:**

Three eligible RCTs were identified. In ADHD+AUD (12 weeks, n≈147), atomoxetine significantly improved ADHD symptoms but did not prolong time to relapse; secondary analyses showed fewer heavy-drinking days. In ADHD+CUD (12 weeks, n=38), atomoxetine yielded partial clinical improvement of ADHD with no differences in cannabis use (days of use, toxicology, craving). In adults receiving methadone (OUD; 53% with CUD), bupropion SR (12 weeks, n=98; comparator arm with methylphenidate) was not superior to placebo for ADHD symptom reduction or cocaine use, with a high placebo response. No RCTs of guanfacine, clonidine, or viloxazine were found. Non-stimulants were generally well tolerated; no signals of misuse or diversion were reported.

**Conclusions:**

Non-stimulant medications alleviate ADHD symptoms in adults with SUD but show no consistent benefits on SUD outcomes. Atomoxetine has the most supportive data, while bupropion shows limited efficacy. Given the small, heterogeneous evidence base, larger and longer RCTs are warranted. The favorable safety profile supports non-stimulants as reasonable first-line options when stimulant misuse is a concern.

**Disclosure of Interest:**

None Declared

